# Supplementary material for: Defining Mechanistic Links Between the Non-Coding Variant rs17673553 in CLEC16A and Lupus Susceptibility
Source: Int J Mol Sci. 2025 Jan 1;26(1):314. doi: 10.3390/ijms26010314 (PMC11720054; doi:10.3390/ijms26010314)

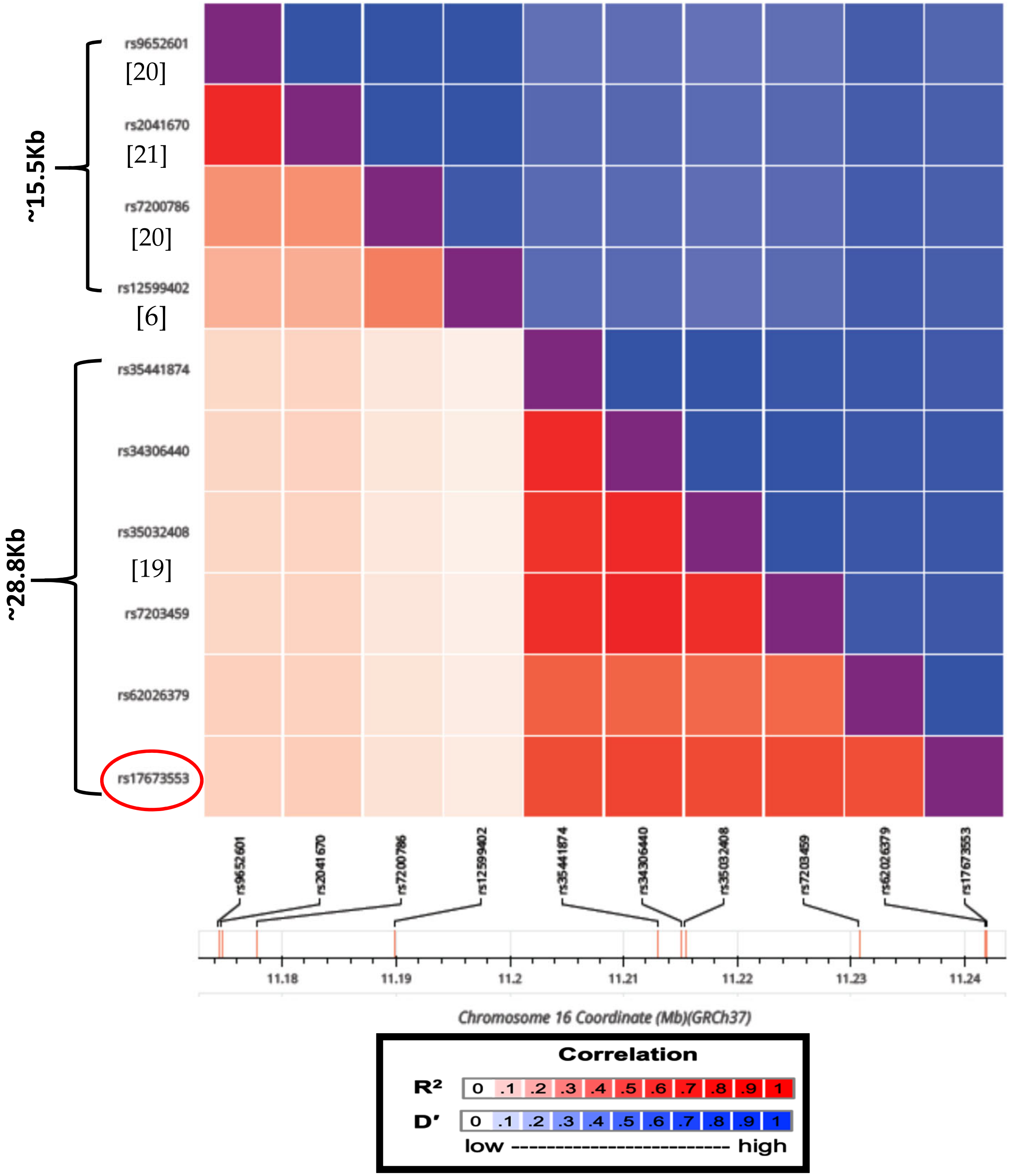

Supplementary Figure S1A: Linkage Disequilibrium (LD) of single-nucleotide polymorphisms (SNPs) around rs17673553 in the 16p13 locus. The LD strengths measured by r2 and D' between SNPs are shown on the lower and upper triangles of the cartoon, respectively. References are shown below SNP names.

rs17673553

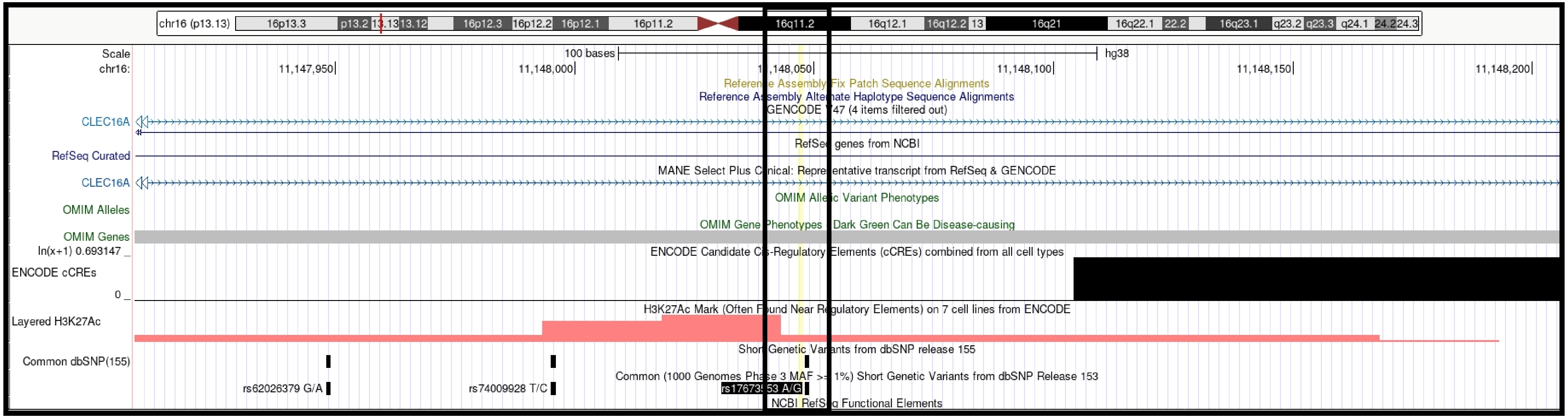

Supplementary Figure S1B: Encode cCREs and Histone marks around rs17673553 region. Bioinformatics analysis of genomic location around rs17673553 (marked in black box) showing the ENCODE cCREs and histone marks (H3K27Ac) in surrounding region.

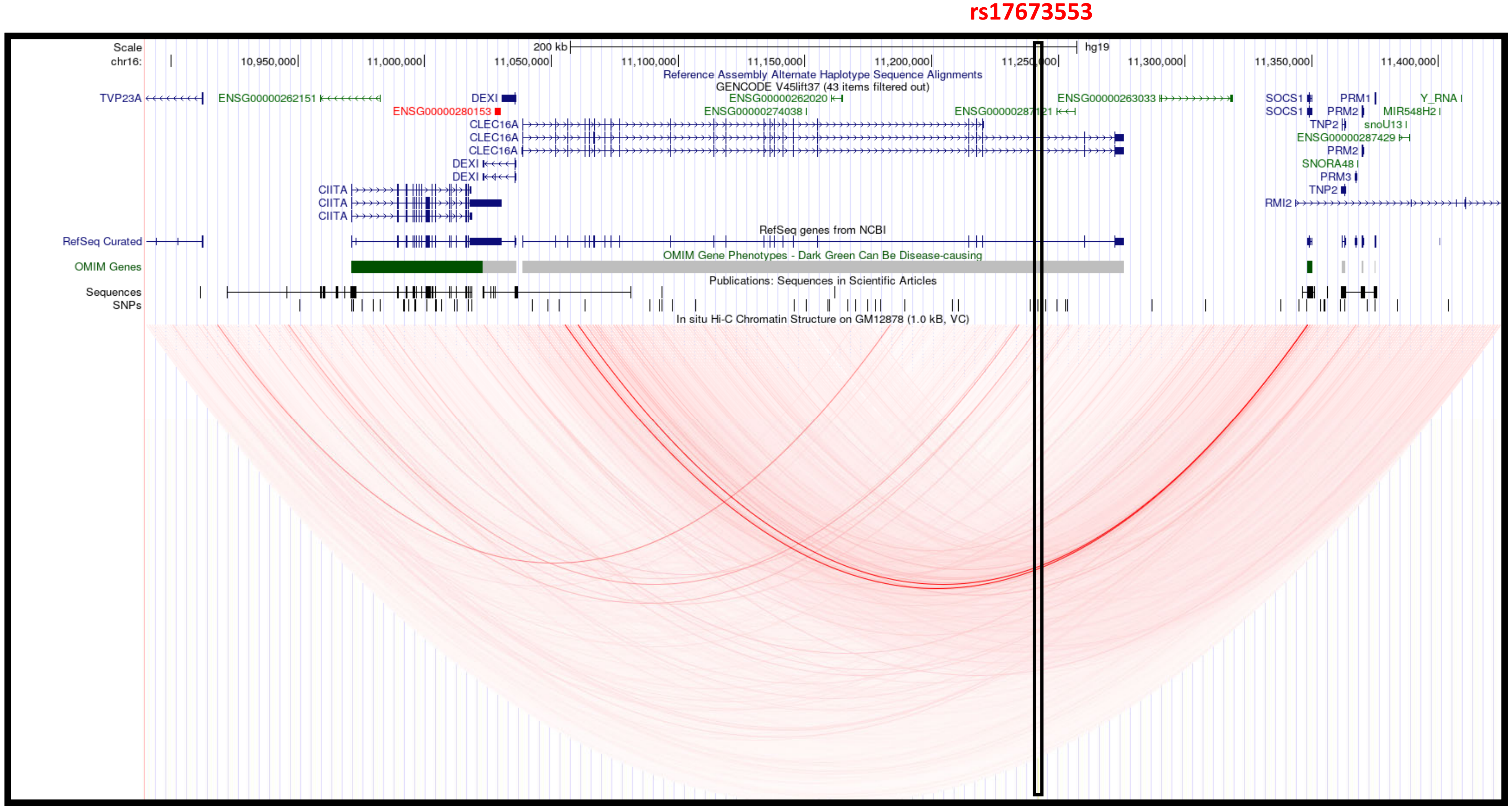

Supplementary Figure S2: Hi-C map of GM12878 cells showing genetic association with the rs17673553 region and values were normalized with Vanilla Coverage. Genomic map showing rs17673553 and nearby genes. Intensity of the red lines indicates interaction confidence.

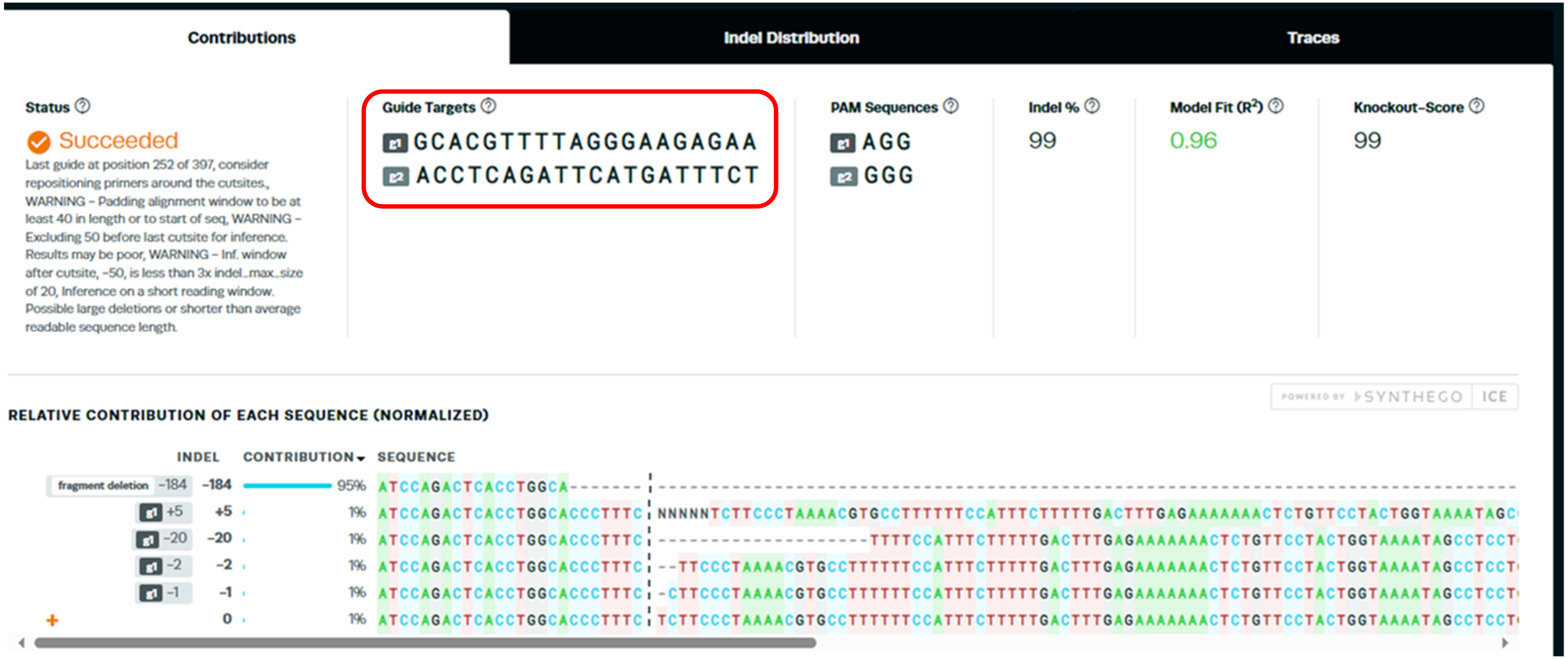

Supplementary Figure S3: ICE analysis of CLEC16A-CRISPR knockout. Sanger sequencing results showing the 95% (row 1) pure clone of *CLEC16A* knockout cell (Namalwa) population when compared with WT cells. Guide RNAs used for knockout are indicated in the red rectangle.

Supplementary Figure S4A: rs17673553 mutates a universally conserved base in binding motifs for GATA2/3/4/6 and TRPS1. The ancestral risk allele A produces more favorable motifs for all TFs. Motifs for POU2F3/POU5F1 are directly adjacent to the SNP. Green highlights bases where the consensus motif is present.

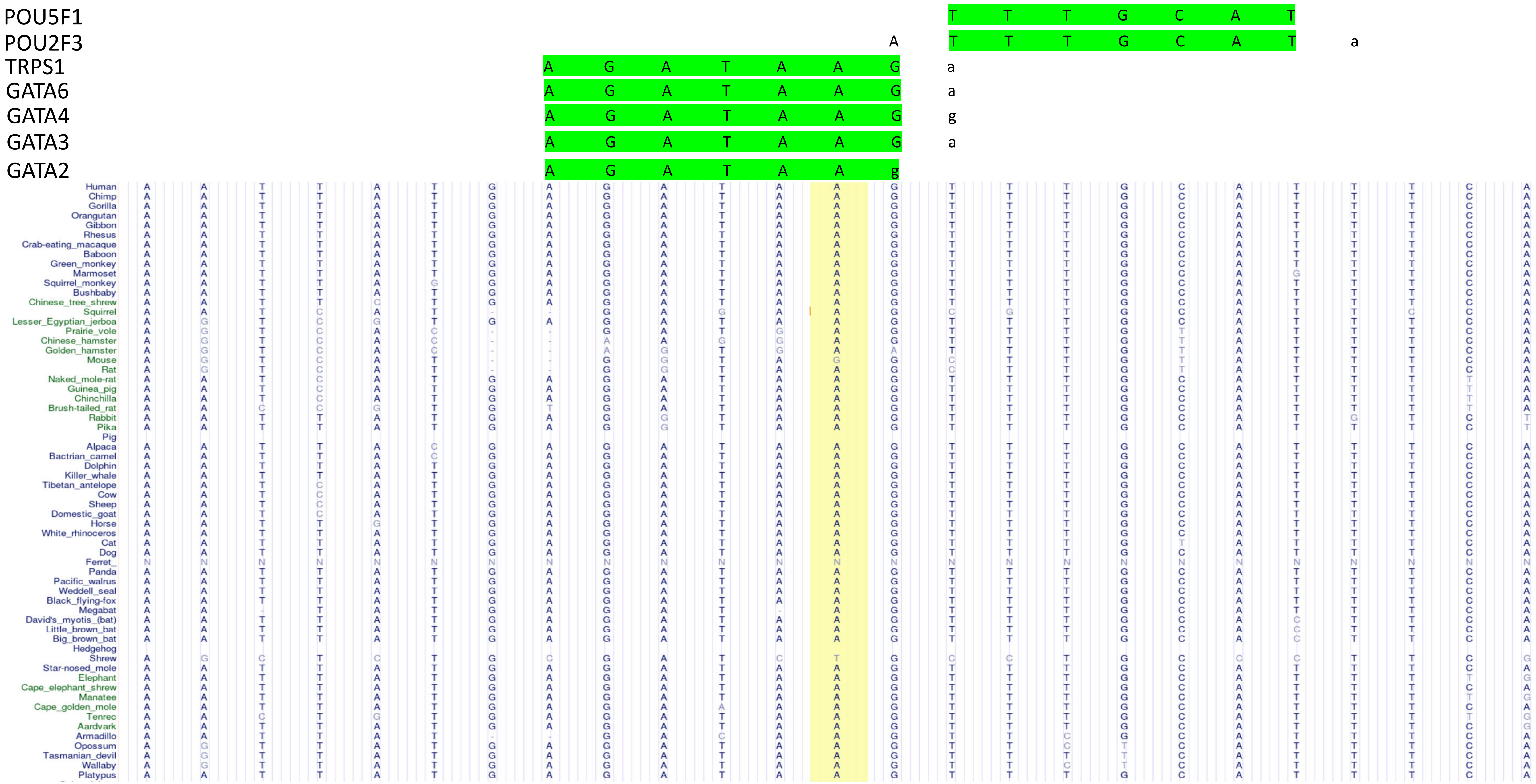

Supplementary Figure S4B: Motifs for STAT1/3/4, IZKF1, and FLI1::FOXI1 are 10bp away from rs17673553. Green highlights bases where the consensus motif is present.

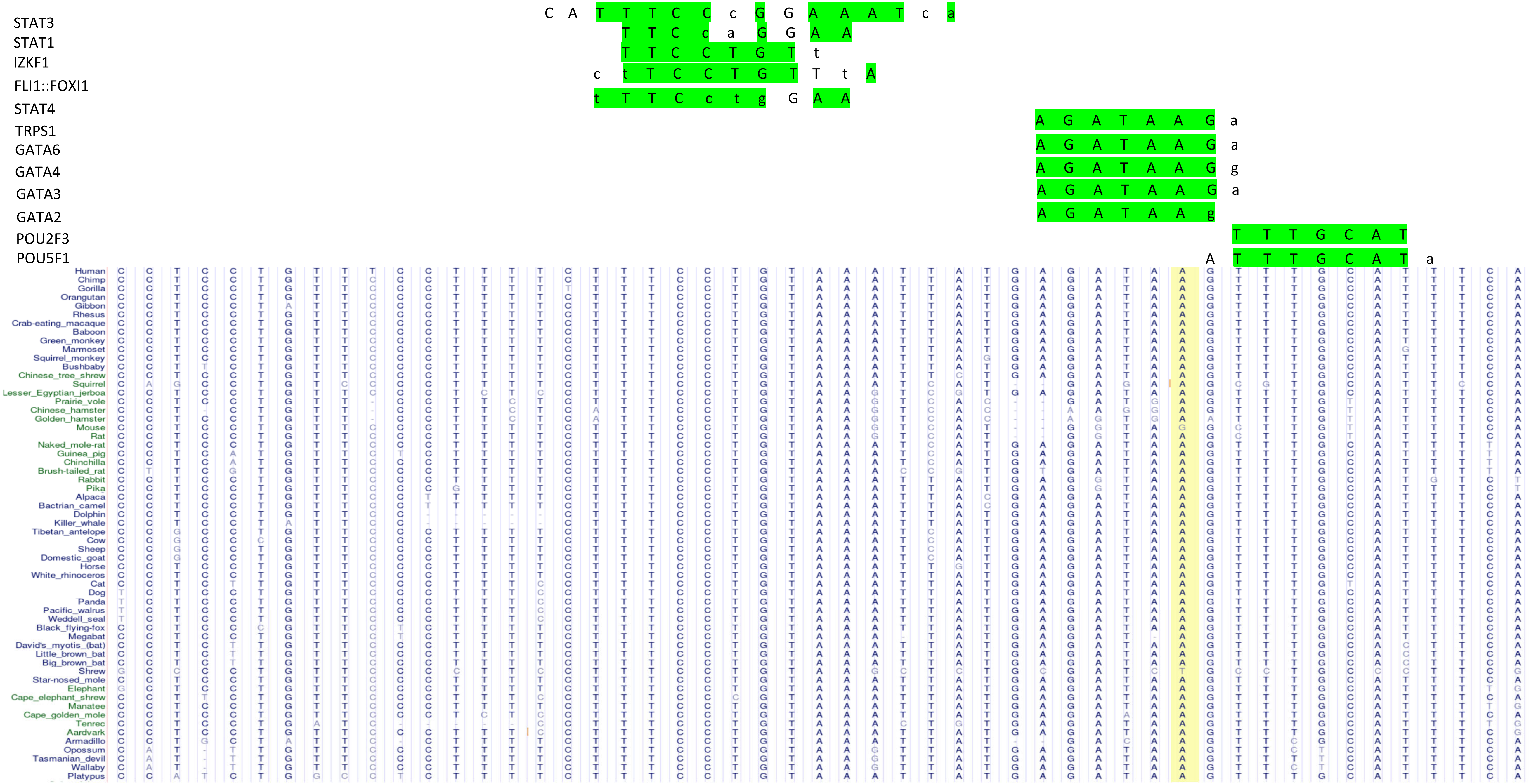

Supplement: Supplementary file 1 [file ijms-26-00314-s001.zip › Supplementary Figures.pdf]
